# Draft Genome Sequences of Two Drug-Resistant Isolates of Pseudomonas aeruginosa Obtained from Keratitis Patients in India

**DOI:** 10.1128/genomeA.01404-14

**Published:** 2015-01-08

**Authors:** Ramesh K. Aggarwal, Chhavi Dawar, Satrupa Das, Savitri Sharma

**Affiliations:** a Centre for Cellular & Molecular Biology (CSIR-CCMB), Tarnaka, Hyderabad, India; b Brien Holden Eye Research Centre, Hyderabad Eye Research Foundation, L.V. Prasad Eye Institute (LVPEI), L. V. Prasad Marg, Banjara Hills, Hyderabad, India

## Abstract

We report here the draft genomes of two drug (fluoroquinolone)-resistant clinical isolates of Pseudomonas aeruginosa obtained from the corneal scrapings of keratitis patients from India. The two annotated genomes are 6.31 Mb and 6.41 Mb in size. These genomes are expected to facilitate the identification and understanding of the genes associated with acquired multidrug resistance.

## GENOME ANNOUNCEMENT

Pseudomonas aeruginosa is a Gram-negative bacterial pathogen associated with a variety of infections (1). The emergence of multidrug resistance in P. aeruginosa isolates has been increasingly documented worldwide. Fluoroquinolones are the most potent agents for the treatment of P. aeruginosa infections (2). However, in recent years, a number of clinical P. aeruginosa isolates have shown reduced susceptibilities or resistances to fluoroquinolones (3, 4). Here, we report draft genomes of two resistant isolates (P2-L230/95 and P7-L633/96) obtained from the corneal scrapings of keratitis patients seen and treated at the L. V. Prasad Eye Institute, Hyderabad, India.

The availability of whole-genome information of pathogens can help in unraveling the genetics/mechanism(s) of acquired drug resistance. Thus, we performed the whole-genome sequencing (WGS) of the two resistant isolates using the Illumina HiSeq and Roche 454 (FLX Titanium) platforms. The isolate identity was confirmed to the species level by typing the 16S rRNA. The reads from Roche 454 (95,836 reads for P2 and 204,602 reads for P7) and Illumina (1,781,644 reads for P2 and 1,271,248 reads for P7) were assembled initially using the GS *de novo* Assembler (version 2.8) and A5-MiSeq assembly pipeline, respectively (5). The resulting contigs were then used to assemble the draft genomes of the two isolates using the Contig Integrator for Sequence Assembly (CISA) of bacterial genomes (6). The draft genome assembly of P2-L230/95 is 6.31 Mb, comprising 110 contigs (range, 4,197 to 371,711 bp in size), with an average length (*N*_50_) of 95,132 bp and 65.70% G+C content. Similarly, the P7-L633/96 genome has 72 contigs (range, 924 to 671,326 bp), with a genome size of 6.41 Mb, an *N*_50_ of 108,322 bp, and 65.97% G+C content. Both genomes were submitted to Rapid Annotations using Subsystems Technology (RAST) and the NCBI Prokaryotic Genome Annotation Pipeline (PGAP) (http://ncbi.nlm.nih.gov/genomes/static/Pipeline.html) for annotation.

In total, 5,823 protein-coding sequences and 63 RNA-coding genes in P2 and 5,949 protein-coding sequences and 60 RNA-coding genes in P7 were annotated using the NCBI annotation pipeline. The RAST server gave comparable results covering 541 subsystems in P2 and 537 in P7. Under the virulence, disease, and defense subsystems in the RAST annotation, the subcategories of resistance to antibiotics and toxic compounds had 121 and 135 genes annotated in P2 and P7, respectively. The alterations in the quinolone resistance-determining regions within topoisomerase II (GyrA and GyrB subunits) and topoisomerase IV (ParC and ParE subunits) are the major mechanisms for fluoroquinolone resistance in Gram-negative bacteria, in addition to a decreased accumulation of fluoroquinolones due to the impermeability of the membrane and/or overexpression of the efflux pump system (7–10). Thus, comparative studies were done using the P. aeruginosa PAO1 genome (GenBank accession no. NC_002516.2) to examine topoisomerase II, topoisomerase IV, and two efflux pump regulatory genes, *mexR* and *nfxB*, to identify putative mutations that might have led to fluoroquinolone resistance. Indeed, we observed several mutations specifically in *gyrA, gyrB*, and *parC* that were reported earlier in drug-resistant strains (11). It is expected that the genomes described here will facilitate detailed studies to identify/elucidate mutation(s)/possible mechanism(s) of acquired drug resistance and better drug discovery.

### Nucleotide sequence accession numbers.

This whole-genome shotgun project has been deposited at DDBJ/EMBL/GenBank under the accession numbers JMBR00000000 for P2-L230/95 and JMBS00000000 for P7-L633/96. The version described in this paper is the second version, with accession numbers JMBR02000000 and JMBS02000000, respectively.
